# The Effect of Ti Particles Addition on the Microstructure and Mechanical Behavior of Mg AZ31/Al 6082 Composite Sheets

**DOI:** 10.3390/ma16072844

**Published:** 2023-04-03

**Authors:** Xiaolei Ai, Yuehui Dang, Xianquan Jiang, Xiaowei Feng, Ruihao Zhang, Yuhe Tian, Jiangyang Yu, He Peng, Rui Hong, Bo Feng, Kaihong Zheng, Fusheng Pan

**Affiliations:** 1 School of Materials and Energy, Southwest University, Chongqing 400715, China; 2 Guangdong Provincial Key Laboratory of Metal Toughening Technology and Application, Institute of New Materials, Guangdong Academy of Sciences, Guangzhou 510650, China; 3 College of Engineering and Technology, Southwest University, Chongqing 400715, China; 4 College of Materials Science and Engineering, Chongqing University, Chongqing 400045, China

**Keywords:** Ti particles, Mg/Al composite, hot rolling, microstructure, mechanical behavior

## Abstract

In this study, Ti particles reinforced Mg AZ31/Al 6082 composite sheets were successfully prepared by hot rolling, with the aim of revealing the effect of Ti particles addition on the mechanical behavior and microstructure of Mg AZ31/Al 6082 composite sheets. The results showed that Ti particles were uniformly distributed at the interface of the Mg/Al-Ti composite sheets, which could greatly reduce the amount of Mg-Al intermetallic compounds during annealing treatment. Compared to the Mg/Al sheet, the tensile strength and elongation of the Mg/Al-Ti sheet could be improved simultaneously after the annealing treatment. Ti particles addition hardly affected the grain size, texture type, and tensile fracture morphology of the Mg layer and Al layer in the composite sheets before and after annealing. This present study provides a new perspective on the mechanical behavior and microstructure of Mg/Al composites through the addition of metal particles.

## 1. Introduction

In recent years, Mg and its alloys have been increasingly used in automobile, aerospace, and 3C industries, due to their low density and high specific strength [1,2,3,4,5,6,7,8]. However, the applications of these Mg alloys have been extremely limited because of their low strength, low formability, and poor corrosion resistance at ambient temperature [9,10,11,12]. As another lightweight material, Al and its alloys exhibit an outstanding corrosion resistance, mechanical performance, and formability [13,14]. Bimetal composites have the performance advantages of two metals and can effectively adjust the performances including strength, conductivity, and formability [15,16]. Therefore, Mg/Al composites have attracted more attention in lightweight fields due to their desirable performance [15,17,18,19,20,21,22].

Various Mg/Al composite sheets have been successfully prepared by rolling bonding [23,24,25], friction stir welding [26,27], explosive welding [28,29], co-extrusion [11,15,30], and diffusion bonding [31,32], etc. For example, explosive welding is more beneficial to join the two metals whose properties differ greatly, but there is some danger in its experimental process [28,29]. Co-extrusion can improve the formability of a composite sheet, but its preparation process is complicated [11,15,30]. Among these techniques, rolling bonding is the most effective method for industrial applications because of its low production cost, good quality, and easy implementation of mass production. Previous publications have reported that Mg-Al intermetallic compounds (IMCs) were generated easily at the interface of the Mg/Al composites during the hot process and subsequent annealing treatment [15,18]. However, these brittle IMCs can greatly affect the mechanical properties of the composites. In our recent publications, the results showed that a very thick diffusion layer (about 300 μm), constituted of abundant Mg-Al IMCs, greatly weakened the mechanical properties of the composites [18,20]. Therefore, how to restrain these harmful Mg-Al IMCs is an important research hotspot in the study of Mg/Al composites.

Up until now, some publications have reported that the introduction of a metal interlayer (such as Zn, Ag, Ni, and Cu, etc.) between the Mg and Al can effectively reduce the amount of Mg-Al IMCs. For instance, Zhao et al. added a Zn interlayer between Mg AZ31 and Al 6061 to eliminate the Mg-Al IMCs in the Mg/Al dissimilar metal joints, which were prepared by diffusion bonding, and the shear strength increased to 83 MPa [33]. Li et al. investigated the effect of a Ni coating on the mechanical performances of Mg AZ91D/Al A356 that were fabricated by compound casting, and the results showed that the Ni coating could improve the shear strength [34]. Gao et al. investigated the laser welding of Mg/Al using a Ti interlayer, and it was shown that the shear strength was improved to 78 MPa by Ti foil [35]. The above researches showed that an appropriate middle interlayer could effectively reduce the amount of Mg-Al IMCs at the interface of a Mg/Al composite, and greatly increase the joint strength of interface. However, there are few studies about the influence of Ti particles on the mechanical properties, texture, and microstructure of Mg/Al composites.

In this study, Mg/Al composite sheets containing Ti particles were fabricated by hot rolling. The microstructures and mechanical performances of the as-rolled and subsequently annealed Mg/Al composite sheets with Ti particles were systematically investigated. In the meantime, the influences of this Ti particles addition on the interface structure and fracture mechanisms of the as-rolled and subsequently annealed Mg/Al sheets were also addressed. This present study provides a new perspective on the microstructure and mechanical performance of Mg/Al composites with an addition of metal particles.

## 2. Materials and Methods

### 2.1. Preparation of Composite Sheets

**Figure 1:**
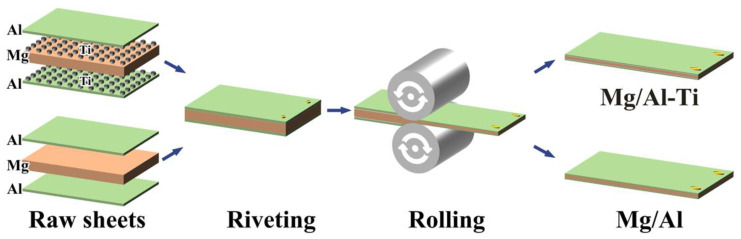
Schematic diagram showing the fabrication of composite sheets by hot rolling.

The materials that were used in the present study were commercial Ti particles, Al 6082 sheet, and Mg AZ31 sheet. The high-purity spherical Ti particles (TA0, Grade 1), with an average size of 100 μm, were supplied by Nantong Jinyuan intelligent company, China. The Al 6082 sheet (thickness of 1 mm) and Mg AZ31 sheet (thickness of 4 mm) were supplied by Shenzhen Shunjinda company, China. The Mg and Al sheets were cut into widths of 125 mm and lengths of 250 mm. The surfaces of the raw sheets were polished with a steel brush. As seen in Figure 1, the Mg and Al sheets were stacked by sequence of 6082/AZ31/6082, and 20 g of the Ti particles were spread evenly between the AZ31 sheet and 6082 sheet. The stacked sheet was placed in an air furnace at 400 °C for 0.25 h and subsequently rolled by a single pass, using a rolling speed of 2 m/min. In order to effectively inhibit the oxidation of the Ti particles, sulfur powder was placed in a muffle furnace to consume the oxygen in the furnace. The final rolled composite sheet (the designated Mg/Al-Ti) had a thickness of 2.7 mm along the normal direction (ND). For comparison, a Mg/Al composite sheet without Ti particles addition was also prepared using the same hot rolling conditions. In order to reveal the influence of the Ti particles on the interface structure of the Mg/Al composite sheets, a portion of them were annealed at 400 °C for 3 h and 10 h, respectively.

### 2.2. Mechanical Tests and Microstructure Measurement

The tensile mechanical performances of Mg and Al raw sheets, Mg/Al and Mg/Al-Ti composite sheets along the rolling direction (RD), and the transverse direction (TD) at room temperature were measured by a universal tester, and the tensile strain rate was 10^−3^ s^−1^. The samples for the tensile tests had a gauge length of 25 mm and width of 5 mm. Each tensile test was repeated three times.

The microstructure and element distribution near the interface of the composite sheets were characterized by a scanning electron microscope (SEM) that was equipped with energy dispersive spectroscopy (EDS). The samples for the SEM were mechanically ground with SiC sand papers, and the measured surface was the RD-ND plane. Inverse pole figure and pole figure were conducted on a Zeiss Gemini 300 SEM with the HKL Channel 5 system, and the measured surface was the RD-TD plane.

## 3. Results and Discussion

### 3.1. Microstructure

**Figure 2:**
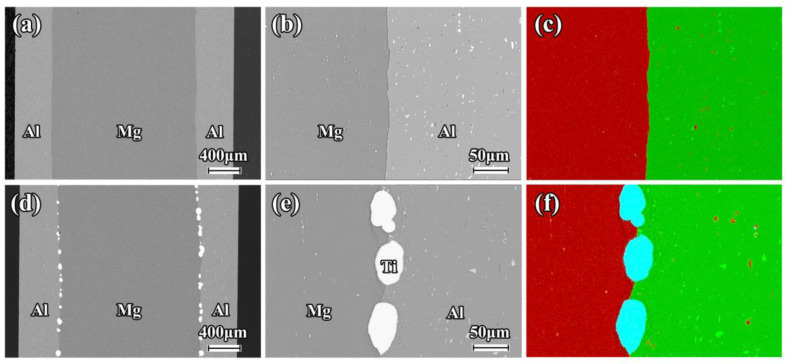
Low magnification and high magnification cross-sectional SEM micrographs of composite sheet: (**a**–**c**) Mg/Al, and (**d**–**f**) Mg/Al-Ti. EDS mapping showing the element distribution of Al (green points), Mg (red points), and Ti (blue points).

**Figure 3:**
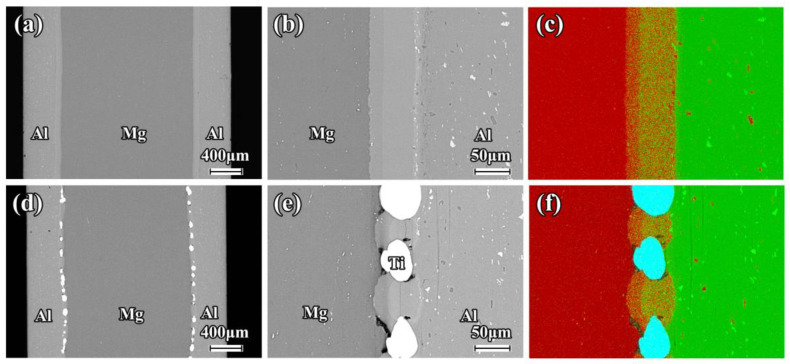
Low magnification and high magnification cross-sectional SEM micrographs of composite sheet after annealing at 400 °C for 3 h: (**a**–**c**) Mg/Al, and (**d**–**f**) Mg/Al-Ti. EDS mapping showing the element distribution of Al (green points), Mg (red points), and Ti (blue points).

**Figure 4:**
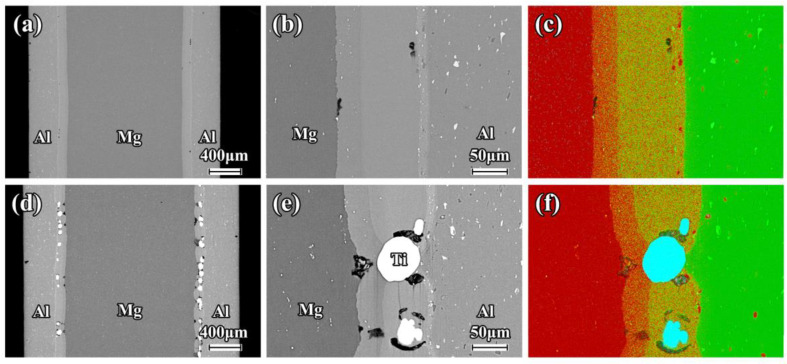
Low magnification and high magnification cross-sectional SEM micrographs of composite sheet after annealing at 400 °C for 10 h: (**a**–**c**) Mg/Al, and (**d**–**f**) Mg/Al-Ti. EDS mapping showing the element distribution of Al (green points), Mg (red points), and Ti (blue points).

The SEM images and EDS mapping of the as-rolled composite sheets near the interface are shown in Figure 2. A good metallurgical bonding condition is clearly revealed in the Mg/Al and Mg/Al-Ti sheets after the hot rolling. There is no obvious diffusion layer close to the interface in these composite sheets. It can be seen that the Ti particles are uniformly distributed at the interface of the Mg/Al-Ti sheet (Figure 2d–f). Figure 3 and Figure 4 show the SEM micrographs of the composite sheets after being annealed at 400 °C for 3 h and 10 h, respectively. After the annealing at 400 °C for 3 h, a thick diffusion layer (about 65 µm in thickness) can be clearly seen in the Mg/Al sheet (Figure 3a–c). An obvious thickening of the diffusion layer, with a thickness of about 120 µm, forms at the interface during the annealing at 400 °C for 10 h (Figure 4a–c). The high-magnification view further shows that the diffusion layer is composed of two sub-layers (Figure 3b and Figure 4b). The sub-layer close to the Al side is thicker than the sub-layer close to the Mg side. This transition layer structure is very common at the interfaces of Mg/Al composites during a hot process. Previous publications have shown that the sub-layer close to Al side is mainly composed of Mg_2_Al_3_, and that the sub-layer that is adjacent to the Mg side has abundant Mg_17_Al_12_ [8,36]. Cross-sectional micrographs of the Mg/Al-Ti sheet after the annealing at 400 °C for 3 h and 10 h are given in Figure 3d–f and Figure 4d–f, respectively. It can be seen that the Ti particles addition can effectively reduce the quantity of the Mg-Al IMCs. However, compared to the annealed Mg/Al sheet, an obvious diffusion layer still formed in the region without the Ti particles addition to the Mg/Al-Ti sheet after the annealing, and the thickness of the diffusion layer does not obviously change. In addition, compared to the annealed Mg/Al sheet, the interface of the Mg/Al-Ti sheet is more likely to form holes after the annealing treatment, and these holes are mainly due to a certain degree of overburning of the Mg-Al IMCs during annealing at a high temperature.

**Figure 5:**
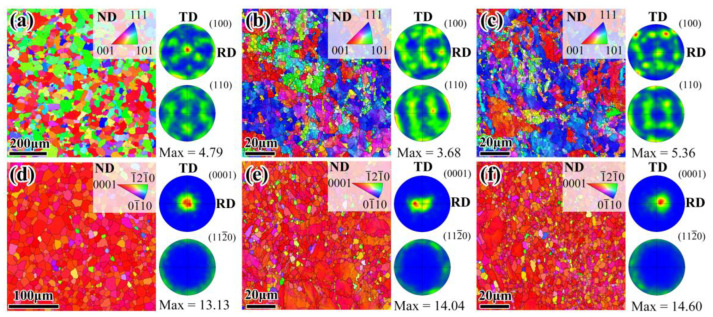
Inverse pole figure and pole figure of the raw sheets and as-rolled composite sheets: (**a**) Al and (**d**) Mg raw sheets, (**b**) Al and (**e**) Mg layers in Mg/Al sheet, and (**c**) Al and (**f**) Mg layers in Mg/Al-Ti sheet. RD, TD, and ND refer to the rolling direction, transverse direction, and normal direction, respectively.

The microstructure and texture of the raw sheets and as-rolled composite sheets are shown in Figure 5. The results show that the raw Mg and Al sheets exhibit a fully recrystallized grain structure, with average grain sizes of about 9 μm and 22 μm, respectively. The raw Al sheet has a representative double texture, with <100> and <101> parallel to the ND (Figure 5a), and the raw Mg sheet exhibits a typical basal texture, with the basal poles being largely parallel to the ND (Figure 5d). Obviously, compared to the raw sheets, both the Mg layer and Al layer of the as-rolled composite sheets have a much finer grain structure. As seen in Figure 5e,f, the Mg layers in the Mg/Al and Mg/Al-Ti sheets contain average grain sizes of 2.5 μm and 2 μm, respectively. The rolled Mg constituent still shows a typical basal texture, with most of its basal pole parallel to the ND, but some <0002> slightly incline toward the RD. However, the texture components of the Al constituent in the composite sheets change, exhibiting a representative double texture with <100> and <111> parallel to the ND (Figure 5b,c). According to the above results, the Ti particles addition hardly affects the grain size and texture type of the as-rolled Mg/Al composite sheet.

**Figure 6:**
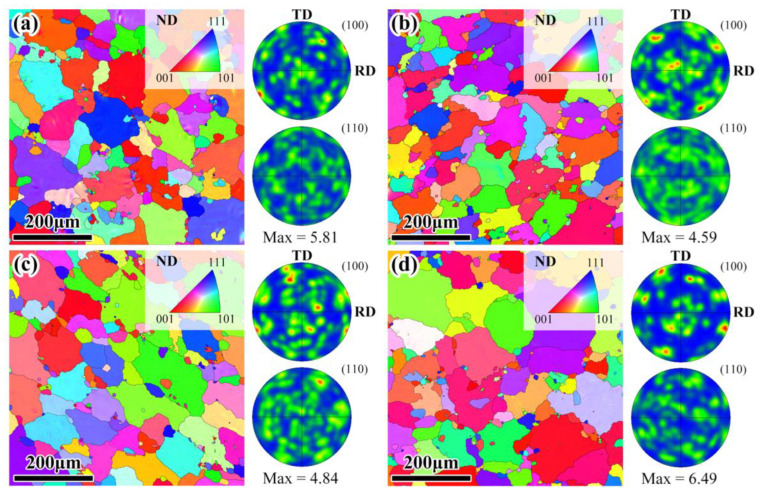
Inverse pole figure and pole figure of the Al layers in composite sheets after annealing: Mg/Al sheet after annealing at (**a**) 400 °C for 3 h, and (**b**) 400 °C for 10 h, Mg/Al-Ti sheet after annealing at (**c**) 400 °C for 3 h, and (**d**) 400 °C for 10 h. RD, TD, and ND refer to the rolling direction, transverse direction, and normal direction, respectively.

Figure 6 shows crystallographic orientation maps of the Al layers in both the Mg/Al and Mg/Al-Ti sheets after being annealed at 400 °C for 3 h and 10 h. The annealed Al layers have a recrystallized microstructure containing a bimodal grain structure. Compared to the Al layer in the as-rolled composite sheets, the grain size of the annealed Al layers increases greatly. Obviously, the average grain size of the annealed Al layer in the Mg/Al sheets hardly change with the Ti particles addition. Compared to the rolled Al layer, the annealed Al layer exhibits a more random texture distribution. Similarly, the texture type of the annealed Al layer hardly changes with the Ti particles addition. Therefore, the above results show that the Ti particles addition has little impact on the grain size and texture type of the Al layer in the annealed Mg/Al composite sheet.

**Figure 7:**
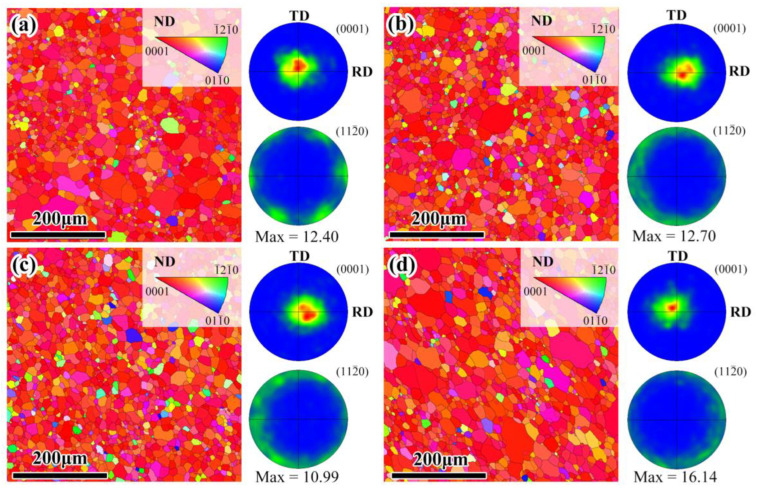
Inverse pole figure and pole figure of the Mg layers in composite sheets after annealing: Mg/Al sheet after annealing at (**a**) 400 °C for 3 h, and (**b**) 400 °C for 10 h, Mg/Al-Ti sheet after annealing at (**c**) 400 °C for 3 h, and (**d**) 400 °C for 10 h. RD, TD, and ND refer to the rolling direction, transverse direction, and normal direction, respectively.

The inverse pole figure and pole figure of the Mg layers in the Mg/Al and Mg/Al-Ti sheets after annealing are given in Figure 7. After annealing at 400 °C for 3 h and 10 h, the grain size of the annealed Mg layers obviously increases, which have average grain sizes of about 13 μm and 14 μm (Figure 7a,b), respectively. In addition, the annealed Mg layers exhibit a typical basal texture of the rolled Mg sheet, with most of the basal poles being parallel to the ND (Figure 7a,b). As seen in Figure 7c,d, the grain sizes of the annealed Mg layers are about 13 μm and 14 μm, respectively. The annealed Mg layers also contain a similar basal texture to the rolled Mg sheet (Figure 7c,d). In consequence, the results show that the grain size and texture type of the annealed Mg layer in the Mg/Al sheets hardly influences by the Ti particles addition.

### 3.2. Mechanical Behavior

**Figure 8:**
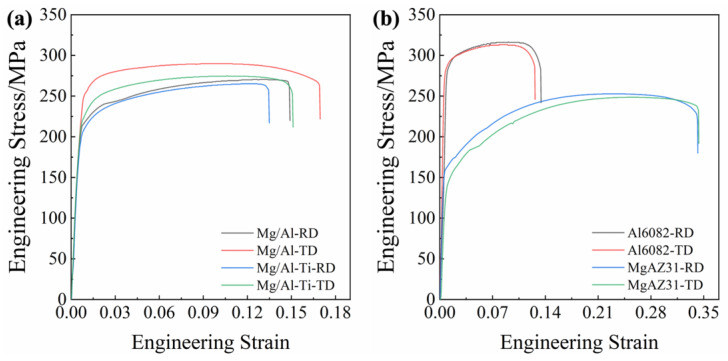
Engineering stress–strain curves under tension along the RD and TD of (**a**) as-rolled composite sheets, and (**b**) raw sheets.

The tensile engineering stress–strain curves of the as-rolled composite sheets and the raw Al and Mg sheets, along the RD and TD, are given in Figure 8. Their mechanical properties are shown in Table 1. The Al 6082 sheet exhibits ultimate tensile strengths of 317 MPa and 313 MPa, and elongations of 13.4% and 12.6% during tension along the RD and TD, respectively. Compared to the 6082 sheet, the Mg AZ31 sheet has lower ultimate tensile strengths (about 249 MPa and 253 MPa) and much higher elongations (about 34.2% and 34.3%). As seen in Figure 8a and Table 1, the rolled Mg/Al-Ti sheet exhibits a lower ultimate tensile strength and elongation than the Mg/Al sheet. Obviously, the Ti particles addition slightly weakens the strength and elongation of the Mg/Al sheet. The main reason for this may be that the interface joint strength between the Mg and Al slightly decreases with the Ti particles addition.

**Figure 9:**
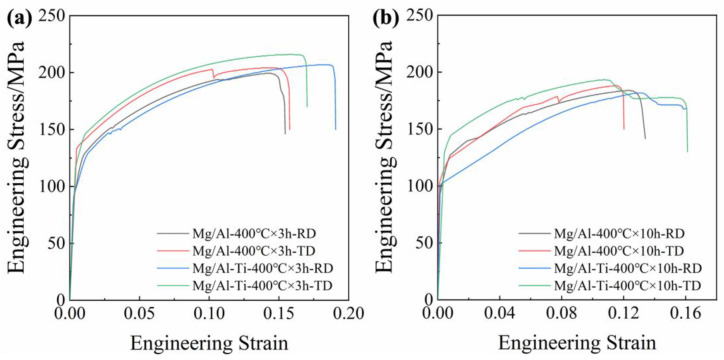
Engineering stress–strain curves under tension along the RD and TD of composite sheets: (**a**) after annealing at 400 °C for 3 h, and (**b**) after annealing at 400 °C for 10 h.

Figure 9 shows the tensile engineering stress–strain curves of the Mg/Al and Mg/Al-Ti sheets after annealing along the RD and TD. Their mechanical properties are given in Table 2. Compared to the as-rolled Mg/Al sheet, the ultimate tensile strength of the annealed Mg/Al sheet is greatly reduced and the elongation shows a decreasing trend. In addition, with the increasing of the annealing time, the elongation of the Mg/Al sheet is decreased significantly. For instance, as seen in Figure 9b and Table 2, after annealing at 400 °C for 10 h, the elongations of the Mg/Al sheet tension along the RD and TD are only 13.4% and 12.0%, respectively. Previous studies have demonstrated that these results might be mainly due to the formation of a large number of Mg-Al IMCs near the interface of the Mg/Al sheet after annealing [8]. However, the annealed Mg/Al-Ti sheet shows a higher UTS and E than the annealed Mg/Al sheet. The primary reason for this may be that the Ti particles can effectively inhibit the formation of Mg-Al IMCs during annealing. Obviously, as seen in Figure 3 and Figure 4, the SEM results strongly support this speculation. Based on the above results, Ti particles addition can simultaneously improve the tensile strength and elongation of a composite sheet after annealing treatment.

**Figure 10:**
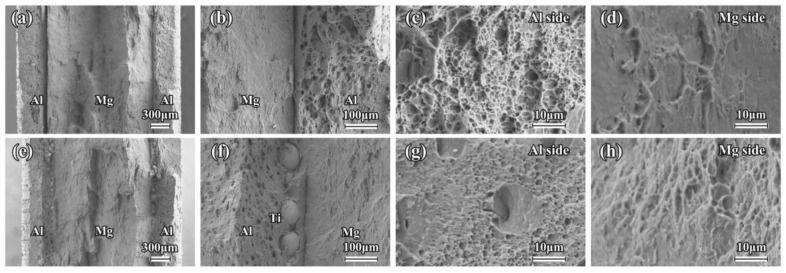
Tensile fracture features of as-rolled composite sheets: (**a**–**d**) Mg/Al sheet, and (**e**–**h**) Mg/Al-Ti sheet.

The tensile fracture micromorphologies of the as-rolled Mg/Al and Mg/Al-Ti sheets are given in Figure 10. There is no severe peeling at the interface near the fracture surface of the as-rolled Mg/Al and Mg/Al-Ti sheets (Figure 10a,b,e,f). It can be seen that the Ti particles are still clearly visible at the interface of the Mg/Al-Ti sheet (Figure 10e,f). The tensile fracture surface of the rolled Al layer has abundant dimples, which shows a typical ductile fracture (Figure 10c,g). As seen in Figure 10d,h, the fracture feature of the rolled Mg layer exhibits a large number of river patterns and some dimples, which is a typical quasi-cleavage fracture morphology. A similar result has also been reported in previous publications [37]. Obviously, the addition of Ti particles hardly affects the tensile fracture feature of the as-rolled Mg/Al composite sheets.

**Figure 11:**
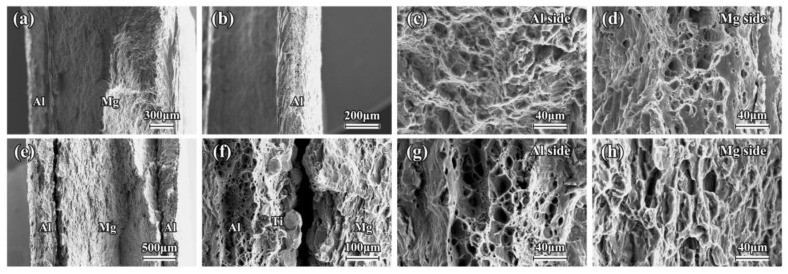
Tensile fracture features of composite sheets annealed at 400 °C for 3 h: (**a**–**d**) Mg/Al sheet, and (**e**–**h**) Mg/Al-Ti sheet.

**Figure 12:**
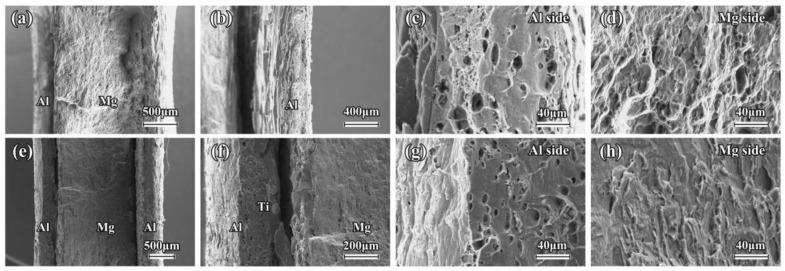
Tensile fracture features of composite sheets annealed at 400 °C for 10 h: (**a**–**d**) Mg/Al sheet, and (**e**–**h**) Mg/Al-Ti sheet.

Figure 11 and Figure 12 show the tensile fracture feature of the composite sheets that were annealed at 400 °C for 3 h and 10 h, respectively. As seen in Figure 11a,b and Figure 12a,b, a severe debonding occurs near the fracture surface in the Mg/Al sheets, and the unilateral Al layer is obviously separate from the Mg layer. This phenomenon may be mainly attributed to the formation of a large number of Mg-Al IMCs near the interface of the Mg/Al sheet after annealing. Usually, a large number of brittle intermetallics, such as Mg_17_Al_12_ and Mg_2_Al_3_, are easily formed at the interface of the Mg/Al composites after annealing. These brittle intermetallics are prone to cracking during loading, eventually resulting in a premature failure of the interface [18,20]. The tensile properties of the annealed Mg/Al sheets also confirm this result. As can be seen from Figure 11e,f and Figure 12e,f, some debonding also occurs at the interface near the fracture surface (Figure 11e,f), but the unilateral Al layer does not separate from the Mg layer, which is obviously different from the annealed Mg/Al sheet. This is mainly attributed to the addition of the Ti particles effectively inhibiting the formation of Mg-Al IMCs, enhancing the interfacial bonding strength of the composites, which greatly affects the tensile fracture mode of the Mg/Al sheets after annealing.

As seen in Figure 11c,d,g,h, the annealed Mg and Al layers of the composite sheets also exhibit a typical quasi-cleavage fracture and ductile fracture morphology, respectively. Interestingly, after being annealed at 400 °C for 10 h, the fracture feature of the Al layer exhibits much more river patterns and fewer dimples, which gradually changes from a ductile fracture to a quasi-cleavage fracture (Figure 12c,g). The Mg layer also shows a quasi-cleavage fracture feature (Figure 12d,h). Therefore, the annealing treatment can greatly affect the tensile fracture morphology of the Mg and Al layers in composite sheets. In the present study, the composite sheets, after being annealed at 400 °C for 10 h, have a thick diffusion layer with a thickness of about 120 µm. Generally, this thick diffusion layer is easy to crack during the loading, leading to a premature failure of the composite plate [18]. This is the reason why the fracture feature of the Al layer in the composite sheets changes after being annealed at 400 °C for 10 h. Therefore, according to the above results, the addition of Ti particles can greatly affect the tensile fracture mode of the Mg/Al sheets after annealing, but hardly changes the tensile fracture morphology of the Mg and Al components.

## 4. Conclusions

In the present study, Ti particles reinforced Mg AZ31/Al 6082 composite sheets were successfully prepared by hot rolling. The influence of Ti particles addition on the mechanical behavior and microstructure of Mg AZ31/Al 6082 composite sheets was systematically studied. Several conclusions were reached, and are as follows:

(1) Ti particles are uniformly distributed at the interface of Mg/Al-Ti composite sheets, which can effectively restrain the formation of Mg-Al IMCs during annealing treatment. Compared to the annealed Mg/Al sheet, an obvious diffusion layer is still formed in the region without Ti particles addition to the Mg/Al-Ti sheet after annealing, but the thickness of this diffusion layer does not obviously change.
(2) Compared to the Mg/Al sheet, the Ti particles addition hardly affects the grain size and texture type of the Mg layer and Al layer in the Mg/Al-Ti sheet, before and after annealing. The rolled and annealed Mg layers always exhibit a typical basal texture, with the basal poles being largely parallel to the ND. The rolled Al layer shows a representative double texture, with <100> and <111> parallel to the ND, but the annealed Al layer exhibits a more random texture distribution.
(3) The annealed Mg/Al-Ti sheet shows a higher tensile strength and elongation than the annealed Mg/Al sheet, which reveals that the Ti particles addition can simultaneously improve the tensile strength and elongation of the composite sheet after annealing treatment. Compared to the Mg/Al sheet, the Ti particles addition hardly affects the tensile fracture morphology of the Mg layer and Al layer in the Mg/Al-Ti sheet before and after annealing.

## Figures and Tables

**Table 1:** Tensile properties of the raw sheets and composite sheets along the rolling direction (RD) and transverse direction (TD). YS, UTS, and E represent yield strength, ultimate tensile strength, and elongation, respectively.

Samples	6082		AZ31		Mg/Al		Mg/Al-Ti	
	RD	TD	RD	TD	RD	TD	RD	TD
YS (MPa)	285	276	164	138	217	255	208	226
UTS (MPa)	317	313	253	249	270	290	265	277
E (%)	13.4	12.6	34.2	34.3	14.9	16.9	13.5	15.0

**Table 2:** Tensile properties of composite sheets after annealing at 400 °C for 3 h and 10 h.

Samples	Mg/Al (400 °C × 3 h)		Mg/Al-Ti (400 °C × 3 h)		Mg/Al (400 °C × 10 h)		Mg/Al-Ti (400 °C × 10 h)	
	RD	TD	RD	TD	RD	TD	RD	TD
YS (MPa)	124	134	117	137	124	121	103	137
UTS (MPa)	199	204	207	216	184	188	191	207
E (%)	15.4	15.7	19.0	17.0	13.4	12.0	13.8	16.2

## Data Availability

Not applicable.
